# Empathy in Chinese medical students: psychometric characteristics and differences by gender and year of medical education

**DOI:** 10.1186/1472-6920-13-130

**Published:** 2013-09-23

**Authors:** Deliang Wen, Xiaodan Ma, Honghe Li, Zhifei Liu, Bensong Xian, Yang Liu

**Affiliations:** 1 The Research Centre for Medical Education, China Medical University, 92 North Second Road, Heping District, Shenyang 110001, China

**Keywords:** Medical students, Empathy, Professionalism, Validity and reliability

## Abstract

**Background:**

In recent years in China, the tense physician-patient relationship has been an outstanding problem. Empathy is one of the fundamental factors enhancing the therapeutic effects of physician-patient relationships and is significantly associated with clinical and academic performance among students.

**Methods:**

This cross-sectional study used the JSPE-S (The Student Version of the Jefferson Scale of Physician Empathy) to assess 902 medical students from 1^st^ year to 4^th^ year at China Medical University. The reliability of the questionnaire was assessed by Cronbach’s alpha coefficient. We performed an exploratory factor analysis to evaluate the construct validity of the JSPE-S. Group comparisons of empathy scores were conducted via the *t*-test and one-way ANOVA. Statistic analysis was performed by SPSS 13.0.

**Results:**

The Cronbach’s alpha coefficient was 0.83. The three factors emerging in the factor analysis of the JSPE-S are “perspective taking”, “compassionate care” and “ability to stand in patients’ shoes”, which accounted for 48.00%. The mean empathy score was 109.60. The empathy score of medical students had significant differences between different genders (*p* < 0.05) and academic year level (*p* < 0.05).

**Conclusions:**

This study provided support for the validity and reliability of the Chinese translated version of the JSPE-S for medical students. Early exposure to clinical training and a curriculum for professional competencies help to enhance the empathy of medical students. We suggest that the curriculum within Chinese medical schools include more teaching on empathy and communicational skills.

## Background

There has been long-standing tension in the physician-patient relationship in China following the economic reform of medical practice and the redelivery of health care [1,2]. There are complex reasons for this phenomenon, but one of the most important factors is the lack of communication skills amongst physicians [3]. In 1999, the Accreditation Council of China for Graduate Medical Education implemented a requirement for residency programs, which focuses on “interpersonal and communication skills that result in effective information exchange while working with patients, their families, and other health professionals” [4]. Empathy is an essential component of communication skills [5,6] and a mutually beneficial attribute to the health provider-patient relationship across all health care professions [7,8], and is thus increasingly important.

Commonly, empathy is the ability to understand other people’s experiences, emotions, and feelings [9]. The most widely recognized definition of cognitive empathy within the context of medical practice is that of Hojat and colleagues at Jefferson Medical College who proposed: “Empathy is a predominantly cognitive (rather than emotional) attribute that involves an understanding (rather than feeling) of experiences, concerns and perspectives of the patient, combined with a capacity to communicate the understanding” [10]. According to some studies, empathy scores were significantly associated with ratings of clinical competence. In examining the relationship between empathy scores and academic performance among students at Jefferson Medical College, students with high empathy scores received higher clinical competence ratings [11]. According to the above, it is crucial for medical school educators and administrators to educate students on the importance of empathy as a necessary skill for providing better patient care and as an integral part of professionalism in medicine. Therefore, measuring and learning the level of medical student empathy is becoming increasingly important, because it can provide a reference for educators to implement curricular changes to either slow the decrease in empathy or increase empathy during undergraduate medical education. Many countries have done several relevant researches [11-15], but there was no previous report in China.

The Student Version of the Jefferson Scale of Physician Empathy (JSPE-S) created by the researchers at Jefferson Medical College in the United States was specifically developed as a self-reporting scale for assessment of empathy in medical students [11,16]. This instrument has been distributed to many countries, has shown satisfactory psychometrics, and has illustrated a number of similarities and differences among the countries [17-19]. Thus, conducting the JSPE-S on medical students and analyzing their cognitive empathy levels is very meaningful in China, where the health care system is somewhat different than in western countries.

The purpose of this study was to examine the psychometric properties of the JSPE-S among a sample of Chinese medical students. We also investigated the primary levels of empathy of the medical students and analyzed group differences.

## Methods

### Participants

Most medical schools in China follow a traditional curriculum, with 5 year bachelor programs for different specialties. Medical training is always divided into 2 years of basic sciences, 2 years of clinical medicine, and 1 year of internship. In this study, the participants were from years 1 to 4 of medical school; fifth year students were excluded. This is due to fifth year students undergoing internships have been dispersed to different regions for practice, making it hard to collect enough effective samples from students in the fifth year of study.

The Chinese student version of the JPSE-S was distributed to 902 clinical medicine students from China Medical University in September 2011. A total of 820 volunteers completed the JSPE-S. Participation was completely voluntary, and students were not compensated for their participation.

### Instrument

The student version (S-Version) of the JSPE was used in this study. It contained 20 items, and each item was answered on a seven-point Likert-type scale. In order to reduce the confounding effect of a response pattern known as the acquiescence response style, half of the items were positively worded (items 2, 4, 5, 9, 10, 13, 15, 16, 17, 20) and directly scored from 1 (strongly disagree) to 7 (strongly agree). The others were negatively worded (items 1, 3, 6, 7, 8, 11, 12, 14, 18, 19) and scored in reverse from 1 (strongly agree) to 7 (strongly disagree). The total score was obtained by summing all items (scores can range from 20 to 140), with higher values indicating a higher level of empathy.

### Procedure

The JSPE-S was first translated into Chinese, and then back-translated into English by two bilingual researchers for medical education at China Medical University to ensure the accuracy of the translation [20,21]. The Chinese student version was used in this study.

The survey was anonymous and voluntary, and informed consent was obtained. The purpose of the study was explained to the participants, and they were asked to provide information on their gender, age, and medical school year of study.

### Data analysis

The missing data were replaced with the median values of the relevant items. However, questionnaires missing information on three or more items were considered ineffective and excluded from subsequent analysis. The data was analyzed using descriptive statistics in this study. Principal component factor analysis with varimax rotation was used to search for the underlying factor structure of the Chinese version of the JSPE-S. The Cronbach’s alpha coefficient was calculated to assess the internal consistency aspect of the reliability of the questionnaire. Furthermore, empathy scores were compared between male and female students by using a *t*-test, and variance analysis was used to examine the differences in age and differences in academic year levels. The SPSS program version 13.0 was used for statistical analyses of the data, and a *p*-value of less than 0.05 was considered significant.

### Ethical approval

The study was approved by the Institutional Review Board from China Medical University. The work was carried out in accordance with the Declaration of Helsinki. Students included in the study gave their oral informed consent. There was no potential harm to participants, and anonymity was maintained.

## Results

Responses were received from medical students from four academic year levels in China Medical University. Of the 902 medical students who received the JSPE-S, 820 completed and returned the survey. 753 of the 820 completed surveys were effective, giving an overall effective response rate of 83.5%. The effective response rate of medical students in each academic year was shown in Table 1.

**Table 1:** The Effective response rate of participants

**Academic year**	**Participants number**	**Effective response number**	**Effective response rate**
1^st^ year	211	181	85.8%
2^nd^ year	267	217	81.3%
3^rd^ year	275	249	90.5%
4^th^ year	149	106	71.1%
Total number	902	753	83.5%

The sample included 277 males (36.8%) and 476 females (63.2%). The total gender ratio of the entire medical student population at China Medical University is 60.1% female students to 39.9% male students. The socio-demographics of the student populations at China Medical University generally represent that of most medical institutions in China. 37 item responses were replaced by the median of the relevant items. The replacement rate was less than 0.05. The mean, standard deviation, quartile points of the JSPE-S are presented in Table 2.

**Table 2:** Descriptive statistic for the Chinese version of the JSPE-S

**Statistic**	**Value**
Mean	109.60
Standard deviation	12.09
25th percentile	102
50th percentile (median)	111
75th percentile	119
Possible range	20–140
Actual range	49–140

### Reliability

In our study, the internal consistency reliability of the questionnaire had a Cronbach’s alpha coefficient of 0.83, which was in the acceptable range for educational and psychological testing as suggested by professional testing organizations [22].

### Construct validity

To assess the appropriateness of using principal components analysis in this study, the Kaiser–Meyer–Olkin (KMO) analysis was performed, yielding an index of 0.90. The result for Bartlett’s test of sphericity was 4931 and was highly significant (*p* <0 .05). This information allowed us to identify the factor model using the exploratory factor analysis approach.

Summary results of factor analysis (principal component factor extraction with varimax rotation) of data for the 20 items of the JSPE are reported in Table 3.

**Table 3:** Exploratory Factor Analysis of the Chinese Version of the JSPE-S

	**Component**		
**Item**	**Factor 1**	**Factor 2**	**Factor 3**
16. Physicians’ understanding of the emotional status of their patients, as well as that of their families, is one important component of the physician–patient relationship.	**.758**	.185	.009
15. Empathy is a therapeutic skill without which the physician’s success is limited.	**.750**	.310	-.052
17. Physicians should try to think like their patients in order to render better care.	**.715**	.212	.019
20. I believe that empathy is an important therapeutic factor in the medical treatment.	**.697**	.247	-.021
10. Patients value a physician’s understanding of their feelings which is therapeutic in its own right.	**.669**	.173	.116
13. Physicians should try to understand what is going on in their patients’ minds by paying attention to their nonverbal cues and body language.	**.634**	.278	-.062
4. Understanding body language is as important as verbal communication in physician–patient relationships.	**.624**	.086	.241
2. Patients feel better when their physicians understand their feelings.	**.595**	-.001	.202
9. Physicians should try to stand in their patients’ shoes when providing care to them.	**.566**	-.016	.095
5. A physician’s sense of humor contributes to a better clinical outcome.	**.558**	-.037	.198
18. Physicians should not allow themselves to be influenced by strong personal bonds between their patients and their family members.	-.405	-.028	.196
14. I believe that emotion has no place in the treatment of medical illness.	.299	**.743**	.127
11. Patients’ illnesses can be cured only by medical or surgical treatment; therefore, physicians’ emotional ties with their patients do not have a significant influence in medical or surgical treatment.	.193	**.693**	.070
12. Asking patients about what is happening in their personal lives is not helpful in understanding their physical complaints.	.181	**.675**	.126
8. Attentiveness to patients’ personal experiences does not influence treatment outcomes.	.022	**.633**	.172
7. Attention to patients’ emotions is not important in history taking.	.244	**.586**	.368
1. Physicians’ understanding of their patients’ feelings and the feelings of their patients’ families does not influence medical or surgical treatment.	-.130	**.493**	-.120
19. I do not enjoy reading nonmedical literature or the arts.	.256	**.474**	.182
6. Because people are different, it is difficult to see things from patients’ perspectives.	.040	.151	**.788**
3. It is difficult for a physician to view things from patients’ perspectives.	.095	.261	**.736**
Percentage of variance	24.19%	15.72%	8.09%

Notes: Items are listed by the order of magnitude of the factor coefficients within each factor. Values greater than 0.450 are in bold.

As shown in Table 3, three factors emerged, accounting for a total of 48.00% of the variance. Factor 1, which accounted for 24.19% of the variance, is a major component (grand factor) that can be labeled “perspective taking” based on the content of the ten items with factor coefficients greater than 0.50 . Factor 2, which accounted for 15.72% of the variance, consisted of seven items with factor coefficients greater than 0.47 and is named “compassionate care”. Factors 3, titled “ability to stand in patients’ shoes”, accounted for 9.37% of the variance and consisted of two items.

### Group comparisons

As shown in Table 4, *t*-test (*t* = 5.57, *p* < 0.005) showed a statistically significant difference between genders. Analysis of variance showed that the differences between mean scores from various years of medical school were statistically significant (*F* = 3.08, *p* < 0.05). In addition, we found by conducting multiple comparisons that the mean empathy score of 1st year and 4th year students had a significant difference (*p* = 0.034 <0 .05). However, the differences between age groups (*t* = 1.29, *p* = 0.20) were not statistically significant.

**Table 4:** Group comparison scores of the Chinese version of the Jefferson scale of medical student empathy

**Groups**	**Number**	**Mean**	**SD**	**Statistical significance**
**Gender**				
Male	277	106.29	13.53	*p* < 0.05
Female	476	111.53	10.72	*t*_(753)_ = 5.57
**Age**				
<22 years old	538	114.01	13.08	*p* = 0.20
≥22 years old	215	112.63	13.94	*t*_(753)_ = 1.29
Academic year*				
1^st^ year^a^	181	107.36	13.35	*p* < 0.05
2^nd^ year	217	109.19	11.39	*F*_(3, 753)_ = 3.08
3^rd^ year	249	110.50	10.80	
4^th^ year^b^	106	112.12	13.55	

Notes: A *p*-value of less than 0.05 was considered significant.

* One-way ANOVA. Mean scores followed by the same letter do not differ according to Student–Newman–Keuls test.

## Discussion

Our findings provide evidence in support of reliability and construct validity of the Chinese version of the JSPE-S for assessing empathy among Chinese medical students. In this study, we obtained the mean scores for Chinese medical students’ empathy, and statistically significant differences were observed between men and women and among different academic years.

The number of fourth year students is much less than other year students in this study. Enrollment numbers differ from year to year at China Medical University due to changes in student population numbers and applicant numbers each year in China. This accounts for the fact that the number of fourth year students is much less than other year students. The overall effective response rate of 4th year students was significantly lower than the rest. One possible explanation is that 4th year students beginning their 6-month clerkship rotations would have less time and interest in completing the questionnaire outside their clerkship duties.

The Cronbach’s alpha coefficient in this study (r = 0.83) was similar to those reported for Korean medical students (r = 0.84) and Japanese medical students (r = 0.80) [18,19]. This result indicates that the JSPE-S is internally consistent in Chinese medical students. Factor analysis in Chinese medical students showed a three-factor solution that was somewhat similar to the pattern that emerged for Iranian, Korean and Portuguese medical students [18,23,24]. This also provided support for the construct validity of the Chinese translation of the JSPE-S.

We found that the mean score for Chinese medical students (mean = 109.60, SD = 13.34) was lower than that was reported for American students (mean = 115, SD = 10) [11] but higher than Japanese students (mean = 104.3, SD = 13.1) [19] and Iranian students (mean = 105.1, SD = 12.9) [23]. This may be attributed to cross-cultural differences in social norms, ethnicity, religious beliefs, pedagogical methods, and sex stereotyping, which can influence empathic engagement [13]. The empathy score in 1^st^ year is lowest. Chinese medical students usually came straight from high instead of coming from undergraduate programs like that in the United States. The demographic characteristics of these younger students suggest a relative lack of life experience and may result in the initial lower levels of empathy. In addition, the National College Entrance Examination only includes written theory examinations, and medical universities mainly recruit students who major in natural sciences (including Physics, Chemistry and Biology) through the Chinese National College Entrance Examinations. They focus less on the Social Sciences, which are advantageous in cultivating personal empathy. Taken together, these reasons can indicate why the empathy score if 1^st^ year is lowest which may contribute to the lower mean empathy score.

We obtained a significant difference in empathy scores between different genders, which was in favour of women (*p* < 0.05, *t*_(753)_ = 5.57) over men and is consistent with most other studies [11,18,19,23]. The gender difference in empathy has been attributed to intrinsic factors (e.g. evolutionary-biological gender characteristics) as well as extrinsic factors (e.g. styles in interpersonal care, socialization, and gender role expectations). This explanation has been widely accepted and supported in relevant literature [11,13,19].

Our study compared the mean empathy scores for medical students in four different medical school years. There were statistically significant differences in empathy scores among medical students in different years of medical school (*F* = 3.08, *p* < 0.05), with the first year students having the lowest empathy scores (mean = 107.36) and the fourth year medical students having the highest (mean = 112.12). Our findings regarding enhancement of empathy during medical school in Chinese students are not in agreement with those recently report for American, Iranian, and Korean medical students [18,23,25-27], but are similar with that of Portuguese and Japanese medical students [19,23].

There are some factors that may have contributed to the improvement of empathy in medical students. In recent years, the difficulty in seeking medical service has been a highlighted problem that needs to be solved in China, due to the number of medical workers in large hospitals being unable to match the need of a large number of patients. Thus, the professionalism and empathy received more attention. It raised many issues that caused medical schools to strengthen their medical education reform. Medical curriculum reforms that introduce early exposure to clinical training gave students opportunities to have more interaction between theoretical and practical disciplines through observing real medical practice and empathizing with patient needs. In the summer of 1999, China Medical University began the curriculum reform by taking New Pathway MD program as lessons. This study was conducted in 2011. Thus, all students experience this early exposure to clinical aspects of medical professionalism and competencies every academic year. By seeing more patients with faculties, medical students were able to have more opportunities to be exposed to role models, which almost all research participants stated as the most effective approach to teach empathy. In addition to exposure to role models, students will develop patient relationships over a longer period of time, which is more conducive for enhancing empathy [28-30]. More sessions concerning professional competencies were added, with the intent that the concepts of empathy and respect for patients will be strengthened during this process.

This study has some limitations. Medical students who participated in this survey came from Liaoning province alone, so we cannot claim that our sample was representative of all Chinese medical students. A replication of the study with a larger and more representative sample of Chinese medical students can strengthen our confidence in the external validity and reliability of the findings. The differences in groups may be attributed to cohort effects. Therefore, a study design using longitudinal annual assessment of the cohorts is warranted.

## Conclusions

Our results provide fundamental support for the reliability and construct validity of the Chinese version of the JSPE-S for medical students. Students in different genders and academic years have significant differences in empathy. We suggest that the curriculum within Chinese medical schools include more teaching on empathy and communicational skills.

## Abbreviations

JSPE-S: The Student Version of the Jefferson Scale of Physician Empathy.

## Competing interests

The authors declare that they have no competing interests.

## Authors’ contributions

WDL designed and conceived the study. MXD administered the surveys. LHH and LY participated in the design of the study and wrote the draft of the manuscript. XBS and LZF participated in its design and performed the statistical analysis. All authors have reviewed and approved the text of the manuscript.

## Pre-publication history

The pre-publication history for this paper can be accessed here:

http://www.biomedcentral.com/1472-6920/13/130/prepub
